# The effects of birth spacing on early childhood development in high-income nations: A systematic review

**DOI:** 10.3389/fped.2022.851700

**Published:** 2022-11-25

**Authors:** Gursimran Dhamrait, Tess Fletcher, Damien Foo, Catherine L. Taylor, Gavin Pereira

**Affiliations:** ^1^ Telethon Kids Institute, University of Western Australia, Perth, WA, Australia; ^2^ School of Population and Global Health, University of Western Australia, Perth, WA, Australia; ^3^ School of Psychology, Curtin University, Perth, WA, Australia; ^4^ Curtin School of Population Health, Curtin University, Perth, WA, Australia; ^5^ Wesfarmers Centre of Vaccines and Infectious Diseases, Telethon Kids Institute, University of Western Australia, Perth, WA, Australia; ^6^ Centre for Child Health Research, University of Western Australia, Perth, WA, Australia; ^7^ Centre for Fertility and Health (CeFH), Norwegian Institute of Public Health, Oslo, Norway; ^8^ enAble Institute, Curtin University, Perth, WA, Australia

**Keywords:** interpregnancy interval, school readiness, birth interval, early childhood, child development, cognitive development, birth spacing

## Abstract

**Objective:**

This study aimed to systematically review the literature on the associations between birth spacing and developmental outcomes in early childhood (3–10 years of age). Studies examining the associations between interpregnancy intervals and child development outcomes during and beyond the perinatal period have not been systematically reviewed.

**Methods:**

We searched Ovid/MEDLINE, Global Health, PsycINFO, EMBASE, CINAHL Plus, Educational Source, Research Starters, ERIC, Scopus, PubMed, Social Science Research Network database, and ProQuest's Social Sciences Databases for relevant articles published between 1 January 1989 and 25 June 2021. Studies published in English, conducted in populations residing in high-income countries with any measure of birth spacing, and child development outcomes among children aged <10 years were included. Two authors independently assessed the eligibility of studies and extracted data on the study design, setting and population, birth spacing, outcomes, and results.

**Results:**

The search yielded 1,556 records, of which seven studies met the inclusion criteria. Five of these seven studies used birth intervals as the exposure measure. Definitions of exposure differed between the studies. Three studies reported an association between short birth spacing and poorer child development outcomes, and two studies reported an association between long birth spacing and poorer child development outcomes.

**Conclusion:**

Currently, limited evidence suggests that the adverse effects of sub-optimal birth spacing are observable beyond infancy.

## Introduction

The early childhood period is a time of rapid growth and development of skills and abilities. Early life experiences strongly mediate childhood development, and the development undertaken during this period forms the basis for future achievements. Variations in development and developmental inequalities that occur during this period can significantly impact the later life outcomes of children (1, 2). In particular, children who face adversities, such as unstable housing, marriage breakdown, and poverty, in their early, formative years are at risk of falling short of their potential (3). Furthermore, sociodemographic factors such as maternal age at birth (4–6), maternal reproductive history (7), and socioeconomic status (including parental educational and occupational characteristics) (8–11) can also influence pregnancy and interpregnancy intervals. Thus, it is important to understand and identify how particular influences associated with pregnancy, birth, and childhood impact children's physical, emotional, and educational development (12). Importantly, there is increasing evidence to suggest that many factors associated with child development exhibit influence during the preconception period and their effect can continue throughout the life course (13).

There is robust evidence for the associations between short and long (i.e., sub-optimal) intervals between pregnancies and adverse pregnancy and birth outcomes for mothers and infants (14); yet it is believed that the pathways governing these outcomes are different. Short IPIs and adverse birth outcomes have been interpreted as evidence in support of the *maternal depletion hypothesis*. The definition of maternal depletion syndrome traditionally states that the cumulative effect of successive pregnancies and lactations results in a worsening of maternal nutritional status (15–19). Alternatively, associations between longer IPIs and adverse birth outcomes have been interpreted as support for the physical regression hypothesis, which proposes that maternal physiological processes are primed for fetal growth during pregnancy and gradually decline over time post-delivery, resulting in a loss of beneficial physiological adaptations from the previous pregnancy (20). Sub-optimal birth spacing is associated with an increased risk of a range of perinatal outcomes, including stillbirth, preterm birth, small for gestational age at birth, and low birthweight (15, 17, 21–23). These prenatal and perinatal outcomes have also been established as risk factors for adverse early and middle childhood development outcomes (24–31). Thus, measures of birth spacing, such as birth-to-pregnancy intervals, have been proposed as important modifiable risk factors for adverse maternal and infant health and developmental outcomes (15, 21, 22, 32).

The concept of optimizing birth spacing has been widely discussed in the literature; the World Health Organization recommends interpregnancy intervals (IPIs) of approximately 2–3 years to reduce infant and child morbidity and mortality (33). However, a majority of these recommendations are based on studies from low- and middle-income countries, which might not be relevant for high-income populations, where the changing obstetric profile (increasing maternal age, use of assistive reproductive technologies, and chronic morbidities) is most relevant (33). Furthermore, these recommendations are largely based on studies examining pregnancy and birth outcomes (33).

Currently, there is no standardized definition for short or long IPIs (22). Short IPIs are commonly defined as being <6 months between the previous birth and subsequent conception but have also been defined as less than 3, 6, 9, 12, or even 18 months (15, 17, 18, 34). Long IPIs are usually classified as >23 months between the previous birth and subsequent conception however are more typically defined as an IPI of at least 60 months (15, 17, 18, 34). Historically, the lowest risk of adverse perinatal outcomes has been in IPIs between 18 and 51 months (35). Thus, IPIs are usually classified as ranges, with short IPIs categorized as: <6, 6–11, and 12–17 months, and long IPIs categorized as either 24–59, 60–119, or ≥120 months (13). IPIs of 18–23 months are typically used as a reference category (13).

Whether there is a significant biological risk for adverse pregnancy outcomes associated with pregnancy intervals is important to establish as birth spacing and unplanned pregnancy rates can be controlled. To date, systematic reviews and meta-analyses have reported that sub-optimal IPIs are associated with an increased risk of adverse prenatal and perinatal health outcomes (14, 16, 21, 36, 37); however, these reviews have not examined child development outcomes beyond infancy. Systematic reviews investigating the impacts of IPIs on child development outcomes beyond birth outcomes have focused on neurodevelopmental morbidities and disabilities, including autism spectrum disorder or attention-deficit/hyperactivity problems (38–41). This study aimed to systematically review the literature on the associations between birth spacing and developmental outcomes during early and middle childhood (3–10 years of age) for children without diagnosed developmental disabilities.

## Methods

We conducted a systematic review of the literature related to birth spacing and child development outcomes, as guided by the minimum evidence-based set of items for reporting in systematic reviews outlined in the Preferred Reporting Items for Systematic Reviews and Meta-Analyses (PRISMA) guidelines (42). The systematic review protocol was registered with the National Institute for Health Research International Prospective Register of Systematic Reviews (CRD42020162265).

### Data sources and search strategy

We conducted electronic searches in Ovid/MEDLINE, Global Health, PsycINFO, EMBASE, CINAHL Plus (EBSCO), Educational Source, Research Starters, ERIC, Scopus, PubMed, Social Science Research Network database, and ProQuest's Social Sciences Database (Supplementary Table S1). We included articles published between 1 January 1989 and 25 June 2021, conducted in populations residing in high-income countries. A search strategy was developed using medical subject headings (MeSH) and keywords related to birth spacing and child development outcomes, with a final search strategy for each database implemented (Appendix One). Studies examining childhood development often examine a range of child-, parental- and community-based sociodemographic characteristics (43–45). Thus, this systematic review included MeSH terms and keywords for family size and sociodemographic factors. As recommended (46, 47), we consulted a medical librarian to assist in the development of the main search strategy.

### Study selection

Eligible studies were all observational studies that investigated the effects of an interval preceding the younger sibling's birth on child development outcomes of the younger child for children aged less than 10 years. For this review, we included studies using measures of birth spacing defined as either: (1) interpregnancy intervals, defined as the time between the birth of the older sibling (i.e., the index child) and the conception of the immediately subsequent pregnancy; or (2) birth intervals (or age-gap), defined as the time between the birth of the older sibling and the birth of the immediately subsequent sibling. This review assessed the five development domains most commonly used to define school readiness among children: (1) cognition and general knowledge; (2) language; (3) social knowledge and competence; (4) emotional health; and (5) physical wellbeing and motor development (48).

Articles were excluded based on the following exclusion criteria: (1) articles published prior to 1989; (2) dissertations, conference papers, case studies, editorials, newspaper articles, and other forms of popular media; (3) articles published in languages other than English; (4) articles that contained “child (and/or) infant mortality” in the title; (5) articles for which the age of outcome assessment for the study cohort was more than 10 years of age; and (6) articles for which the study population comprised of children with developmental disabilities, including autism spectrum disorder, intellectual disability, based on the assumption that these outcomes have a large genetic basis.

Previous studies have indicated that puberty onset is associated with divergent trajectories in behavior, emotion, cognition, and brain development for males and females even after accounting for age-based differences (49–52); thus, studies were limited to prepubertal children (i.e., aged less than 10 years). Studies were limited to high-income countries for two reasons. First, birth spacing and child development outcome demographics are different between low-middle-income countries and high-income countries; short birth intervals are more common among women in low-middle-income countries (53). Likewise, a greater proportion of children residing in low-middle-income countries are classified as being at risk of poor childhood development (54, 55). Furthermore, low-middle-income countries have a larger on average rural-urban gap in terms of child development compared with high-income countries (55). Second, the determinants for sub-optimal birth spacing are likely to be different between low-middle-income countries and high-income countries (53, 56). Low-middle-income countries typically have higher fertility rates and higher levels of unmet family planning needs, which leads to shortened intervals between pregnancies (56). Alternatively, in high-income countries, there is an increasing proportion of women delaying the initiation of childbearing until their thirties, primarily for additional education attainment and career progression purposes (57–59). Although higher levels of maternal education are associated with improved child development outcomes (60), it can also impact birth spacing. Delaying childbearing not only impacts the first pregnancy but also subsequent pregnancies are complicated by increasing maternal age and the negative effects of the *biological clock* (61). As a result, women who delay the initiation of childbearing may be inclined to accelerate subsequent pregnancies in order to minimize the declines in fecundability and fertility associated with advancing maternal age, resulting in short IPIs between pregnancies (62).

Studies were screened using a three-stage review approach in which the title, followed by the abstract, and then the full texts were assessed. Screening and article selection was conducted using Covidence systematic review software (63). Two reviewers independently screened and reviewed the titles and abstracts of records retrieved during the search for inclusion criteria. The reviewers then screened and reviewed the full-text articles for eligibility in accordance with the inclusion and exclusion criteria. Studies judged to have met the inclusion criteria by the two reviewers were included in the final review. A third reviewer resolved any conflicts between the two reviewers during each screening and review stage.

### Data extraction

We developed a standardized data collection form to extract information on study characteristics, including study type, geographic location, study population demographics, type of birth spacing measure, categorization of birth spacing measures, child development outcome measures, adjusted odds ratios or relative risk ratios and the associated confidence intervals, and confounding variables. Two reviewers independently extracted information from the included articles.

### Quality assessment and risk of bias assessment

Two reviewers independently assessed the quality of cohort, case-control, and cross-sectional studies using the Newcastle-Ottawa scale to evaluate the risk of bias (Supplementary Tables S3–S5) (64). The Newcastle-Ottawa scale is based on three major components: (1) selection bias (maximum score: 4); (2) comparability of study groups (maximum score: 2); and (3) ascertainment of exposure for case-control and cross-sectional studies or outcome for cohort studies (maximum score: 3) (64). In line with previous studies, Newcastle-Ottawa scale scores were classified into three categories (65–67). Studies scoring: 0–3, 4–6, and 7–9, were considered to have high, moderate, and low risk of bias, respectively. Any conflicts between the two reviewers during the quality assessment process were resolved by a third reviewer.

### Data synthesis and analysis

We developed a narrative description of the study characteristics and results for all the included studies. To generate pooled effect estimates for each outcome, a random-effects meta-analysis using the inverse variance method (68) to explore the associations between interpregnancy intervals and child development outcomes was planned. This was dependent on whether a sufficient number of studies was retrieved using commonly defined exposure and outcome measures. Due to the limited number of studies, publication bias was not examined.

## Results

### Study selection and characteristics

**Figure 1:**
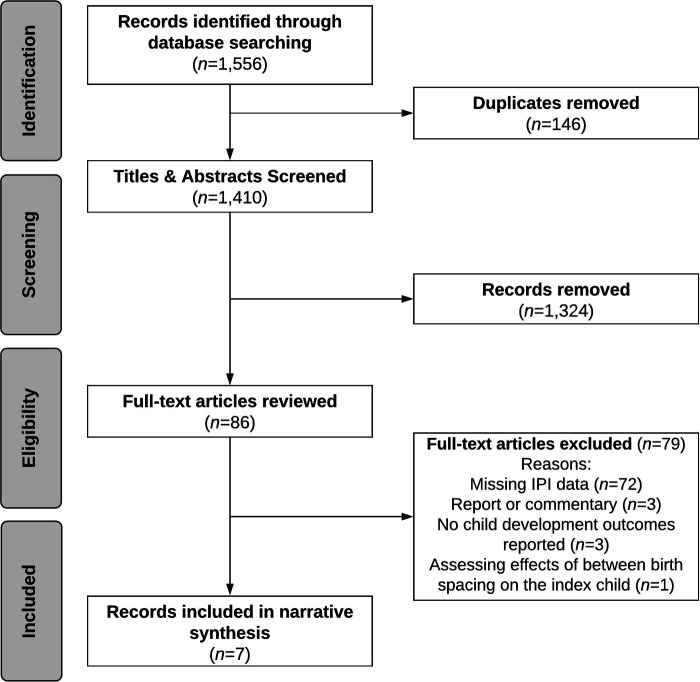
Flow diagram of study selection process for systematic review of the literature on birth spacing and child development outcomes.

The search produced a total of 1,556 records. After removing duplicates (*n* = 146), 1,324 studies were excluded after the initial title and abstract screening (Figure 1). We reviewed 86 full-text articles, of which seven met the selection criteria. Studies were excluded if there was no measure of birth spacing (*n* = 72), were a report or commentary (*n* = 3), did not report on child development outcomes (*n* = 3), and if the effect of birth spacing was assessed for the index child (*n* = 1).

The seven studies had varied methodologies and study outcomes (Table 1). Study periods ranged from 1973 to 2015. The studies were conducted in very different settings—three in the United States, two in Saudi Arabia, one in Australia, and one in France. Two studies used a cross-sectional design (69, 70), four used a retrospective cohort design (8, 71–73), and one used a case-control design (74). Study sample sizes ranged from 536 to 34,574 children. Exclusion criteria varied between the seven studies (Supplementary Table S6); however, all seven studies excluded children from multiple births. One study reported a series of models to adjust the results (73); thus, results from both adjusted models were reported for this study. Due to differences in the categories and reference categories of birth spacing intervals and outcome measures and the limited number of studies on any particular outcome, meta-analyses were not performed.

**Table 1:** Characteristics of studies included in the systematic review.

Author (year) Location	Study design and (sample size)	Study period	Exposure measure: interval category.	Ascertainment of exposure	Outcome measure
Bella and Al-Almaie (2005) Saudi Arabia	Cross-sectional study (*n* = 536). Male children aged 9–10 years	2000–2001	Birth interval: <17 months (lower quartile) and >31 months (upper quartile)	Self-reported by parents	School performance: Highest grade (A) scored in year of study, Highest grade (A) scored in previous year, Teacher-assessed school performance: below average, and Teacher-assessed school performance: above average
Bella et al. (2005) Saudi Arabia	Cross-sectional study (*n* = 546). Male children aged 9–10 years	2000–2001	Birth interval: <13 months (lower quartile), 13-18 months, 19-24 months, and >31 months (upper quartile)	Self-reported by parents	Intellectual ability/general intelligence levels (assessed using the Progressive Raven's Matrices Test)
Dhamrait et al. (2021) Western Australia, Australia	Retrospective cohort study (*n* = 34,574). Children in the first year of full-time school (5 years of age) Mean age category: ≥5 years and 1 month and <5 years and 4 months	2002–2015	Interpregnancy Intervals: <6 months, 6–11 months, 12–17 months, 18–23 months (reference category), 24–59 months, 60–119 months, and ≥120 months	Birth and perinatal records	School readiness (assessed using the Australia Early Development Census (AEDC). Developmental Vulnerability on one or more AEDC domains (DV1). Developmental Vulnerability on two or more AEDC domains (DV2). Developmental Vulnerability in five domains: 1. Physical health and wellbeing, 2. Social competence, 3. Emotional maturity, 4. Language and cognitive skills (school-based), and 5. Communication skills and general knowledge
Havron et al. (2019) France	Retrospective cohort study (*n* = 1,154) Children aged 0–11 years	2003–2006	Age gap (proxy measure of birth interval): continuous variable	Self-reported by mother	Language skills/development [assessed using the NEuroPSYchological Assessment (NEPSY), and Wechsler Preschool and Primary Scale of Intelligence (3rd Ed.) (WPPSI-III)]
Hayes et al. (2006) South Carolina, USA	Retrospective cohort study (*n* = 6,915)	2000	Birth Interval: <24 and ≥24–120 months (including 6-month increment sub-groups)	Medical records	School readiness [assessed using the Cognitive Skills Assessment Battery (CSAB) test]
Nathens et al. (2000) Washington, USA	Case-control study (*n* = 3,145 cases; *n* = 8,371 controls)	1989–1996	Birth Interval: <24, 24–48 months >48 months, Unknown	Medical records	Injury-associated hospitalization or death (ICD-9: unintentional injuries (E800–E869; E880–E929), intentional injuries (E960–E969), and other injuries (E950–E959; E980–E989)
Sujan et al. (2019) USA	Retrospective cohort study (*n* = 5,339)	1973–2008	Interpregnancy Intervals: <12 months, 12 to ≤36 months, >36 months	Self-reported	Language development using the Peabody Picture Vocabulary Test (PPVT) administered biennially from ages 4–12 years. The Digit Span subtest of the Wechsler Intelligence Scales for Children-Revised (WISC-R) and the Maths, Reading and Reading Recognition subtests of the Peabody Individual Achievement Test-Revised (PIAT-R) administered biennially from ages 7–12 years. Externalizing behavioral symptoms (Conduct, Oppositional Defiant and Attention-deficit/hyperactivity) using the Behavior Problems Index, completed by the mother of children aged 4–9 years.

### Exposure assessment

Four studies relied on parental self-report (69–71, 73) and three from medical records for birth spacing measures (8, 72, 74). The categories and reference categories of birth spacing intervals differed across the seven studies. Two studies used IPIs as the measure for birth spacing (8, 73), whilst five studies used birth intervals between siblings as a proxy measure for birth spacing (Table 1). The studies included in this review defined short birth spacing as birth intervals of <13 months (70), <17 months (69), and <24 months (72, 74), or IPIs of ≤12 months (73) and <18 months (8). Likewise, definitions of long birth spacing also varied between the studies included in this review and were defined as birth intervals of 24–48 months (74), >31 months (69, 70), and >48 months (74), or IPIs of >24 months (8) and >36 months (73). Reference categories for birth spacing varied across the seven studies; one study compared children with birth intervals to children with no older siblings, which included first-born children and children without siblings (74).

### Outcome assessment

The studies included in this review measured a variety of different outcomes, including school performance (69), intellectual ability and general intelligence levels (measured using the standardized version of The Standard Progressive Raven's Matrices test; hereafter the Raven's Matrices test) (70), child behavior (measured using the Behavior Problem Index) and development [measured using the Peabody Picture Vocabulary Test (73); the Digit Span subtest of the Wechsler Intelligence Scales for Children-Revised; and the Maths, Reading, and Reading Recognition subtests of the Peabody Individual Achievement Test-Revised] (73), school readiness [measured using the Cognitive Skills Assessment Battery (72), and the Australian Early Development Census (AEDC)] (8), and injury-associated hospitalization or death (Table 1) (74).

### Adjustment variables assessment

Among the seven included studies, a total of 13 maternal, 8 child and 9 sociodemographic characteristics were included as potential confounders (Table 2). Common maternal characteristics included age at the time of delivery (adjusted for in five studies), education (adjusted for in three studies), and smoking/alcohol consumption during pregnancy (adjusted for in three studies); whilst ethnicity, breastfeeding status, marital status and, intelligence quotient (IQ), depression, self-reported delinquency and whether the mother had a miscarriage, stillbirth, or abortion occurred within the focal child's IPI were less commonly controlled. Child characteristics primarily included sex (adjusted for in three studies) and gestational age (adjusted for in two studies), whilst other characteristics such as birth order, ethnicity, and language spoken at home were less commonly controlled (Table 2). Sociodemographic characteristics primarily included household income (adjusted for in two studies), paternal education or occupation status (adjusted for in two studies), and the presence of a younger sibling at the time of evaluation (adjusted for in two studies), whilst other characteristics including socioeconomic status index or housing, the adequacy of prenatal care, paternal age, paternal education, and family composition (whether the family included half-siblings) were less commonly controlled (Table 2).

**Table 2:** Adjustment variables listed in original studies.

Adjustment variables	Author (year)						
	Bella and Al-Almaie (2005)	Bella et al. (2005)	Dhamrait et al. (2021)	Havron et al. (2019)	Hayes et al. (2006)	Nathens et al. (2000)	Sujan et al. (2019)
**Maternal characteristics**							
Age at child's birth	–	–	X	X	X	X	X
Ethnicity	–	–	–	–	X	–	X
Education	–	–	–	X	X	–	X
Marital status	–	–	X	–	X	–	–
Breastfeeding status	–	–	–	X	–	–	–
Smoking/alcohol consumption during pregnancy	–	–	X	X	–	–	X
IQ score	–	–	–	–	–	–	X
Mode of delivery	–	–	X	–	–	–	–
Occupational status	–	–	X	–	–	–	–
Parity	–	–	X	–	–	–	–
Depression	–	–	–	–	–	–	X
Self-reported delinquency	–	–	–	–	–	–	X
Whether a miscarriage, stillbirth or abortion occurred within the focal child's IPI	–	–	–	–	–	–	X
**Child characteristics**							
Gestational age	–	–	X^a^	X	–	–	–
Birthweight	–	–	–	X	–	–	–
Sex	–	–	X	X	–	–	X
Small-for-gestational age	–	–	X	–	–	–	–
Ethnicity	–	–	X	–	–	–	–
Child speaks a language other than English at home	–	–	X	–	–	–	–
Age at time of assessment	–	–	X	–	–	–	–
Birth order	–	–	–	–	–	–	X
**Sociodemographic characteristics**							
Household income	–	–	–	X	–	–	X
Paternal age at child's birth	–	–	–	X	–	–	–
Paternal education or occupational status	–	–	X	X	–	–	–
Adequacy of prenatal care	–	–	–	X	–	–	–
Presence of a younger sibling at the time of evaluation	–	–	–	X	–	–	X
Family composition, whether the family included half-siblings	–	–	–	–	–	–	X
Total number of siblings	–	–	X	–	–	–	–
Remoteness index	–	–	X	–	–	–	–
Socioeconomic status index or housing	–	–	X	–	–	–	–

Each X represents whether an individual criterion is satisfied. Each – represents whether an individual criterion is not satisfied.

^a^ Adjusted for preterm birth.

### School readiness

Two studies examined the relationship between birth spacing and school readiness. Hayes et al. (72) reported that there was a mean difference in the Cognitive Skills Assessment Battery score, a measure of school readiness, between children born after a birth interval of ≥24–120 months and children born after a shorter birth interval (<24 months) (72). Each additional 6-month incremental increase in birth intervals was associated with a decreased risk of the child not being ready for school [adjusted odds ratio (aOR), 0.96; 95% CI, 0.95–0.98] (72). This study also reported that a 6-month reduction in birth intervals had a stronger effect on the school readiness of children born to African American mothers (aOR, 1.50; 95% CI, 1.34–1.78). However, this study also reported that children born to African American mothers were 1.63–1.70 times more likely to not be ready for school compared with children born to White American mothers, irrespective of birth interval.

Dhamrait et al. (8) reported that compared with the reference category (18–23 months), IPIs of <6 months were associated with an increased risk of developmental vulnerability on one or more (aOR, 1.17; 95% CI: 1.08–1.34), and two or more (aOR, 1.31; 95% CI, 1.10–1.54), AEDC domains (8). All IPIs longer than the reference category were associated with an increased risk of children being classified as developmental vulnerability on one or more, and on two or more, AEDC domains (aOR, >1.15) (8).

### Child behavior

Two studies reported on the associations between IPIs and child behavior (8, 73). Sujan et al. (73) used the Behavior Problem Index to assess symptoms of conduct problems, oppositional defiant problems, and attention-deficit/hyperactivity problems. After adjustment for child's sex and birth order, short IPIs (≤12 months) were associated with a reduced risk of oppositional defiant problems (aOR, 0.98; 95% CI, 0.90–0.86), compared with the reference category (IPIs of >12–36 months). After adjustment for the child's sex and birth order, no association was reported between short IPIs (≤12 months) and conduct problems or for attention-deficit/hyperactivity problems. After adjustment for child's sex and birth order, no association was reported between long IPIs (>36 months) and child externalizing behavior. In the fully adjusted models, this study reported no associations between short (≤12 months) and long (>36 months) IPIs and symptoms of conduct problems, oppositional defiant problems, or attention-deficit/hyperactivity problems, compared with the reference category (IPIs of >12–36 months) (73).

Dhamrait et al. (8) assessed the associations between IPIs and the Emotional Maturity and Social Competence domains of the AEDC. This study reported that compared with the reference category (18–23 months), short IPIs of <6 and 6–11 months were associated with an increased risk of being classified as developmentally vulnerable for the Emotional Maturity domain (aOR, ≥1.27) (8). IPIs longer than the reference category were associated with an increased risk of being classified as developmentally vulnerable on both the Emotional Maturity (aOR, ≥1.23) and the Social Competence domains (aOR, ≥1.20) (8).

### Language skills and development

Three studies reported on the associations between birth spacing and language skills and development (8, 71, 73). Havron et al. (71) reported that language skills were lower for children without an older sibling compared with children with an older sibling (Cohen's *d*, −0.17); however, no association was reported between the age gap between siblings and lower language skill scores of the younger sibling [adjusted beta-coefficient (a*β*), −0.035; SD, 0.019]. Sujan et al. (73) reported that children born after short IPIs (≤12 months) had lower standardized scores on measures of spoken word and receptive vocabulary knowledge of a child after adjustment for child's sex and birth order (aOR, 0.82; 95% CI, 0.73–0.92), compared with the reference category (IPIs of >12–36 months). No association between standardized scores on measures of spoken word and receptive vocabulary knowledge of a child and both short IPIs of ≤12 months (aOR, 0.99; 95% CI, 0.90–1.09) or long IPIs of >36 months (aOR, 1.03; 95% CI, 0.92–1.15), compared with the reference category, in the fully adjusted models.

All IPI categories longer than the reference category (18–23 months) were associated with an increased risk of children being classified as developmentally vulnerable on the Language and Cognitive Skills (school-based) domain of the AEDC (aOR, ≥1.25) (8). Compared with the reference category, long IPIs of 60–119 and ≥120 months were associated with an increased risk of children being classified as developmentally vulnerable in the Communication Skills and General Knowledge domain (aOR, ≥1.35) (8).

### Physical health

Nathens et al. (74) assessed the relationship between birth intervals and the risk of injury to the index child. After adjustment for maternal age, this study reported an increased risk of injury for children born after a birth interval of <24 months (aOR, 1.64; 95% CI, 1.44–1.85) and >48 months (aOR, 1.46; 95% CI, 1.30–1.64), compared with children with no older siblings (74). Compared with children with older siblings, but for whom a birth interval could not be determined were also reported to have an increased risk of injury (aOR, 1.47; 95% CI, 1.24–1.75) (74).

Dhamrait et al. (8) examined the associations between IPIs and Physical Health and Wellbeing at school starting age. This study reported that IPIs of <6 months were associated with an increased risk of children being classified as developmentally vulnerable on the Physical Health and Wellbeing domain of the AEDC (aOR, 1.25, 95% CI, 1.06–1.48) (8). Compared with the reference category, long IPIs of 60–119 and ≥120 months were associated with an increased risk of children being classified as developmentally vulnerable in the Physical Health and Wellbeing domain (aOR, ≥1.35) (8).

### School performance

Bella and Al-Almaie (69) reported more children born after long birth intervals (>31 months) achieved the highest grade (grade A) in the current year of study compared with children born after short birth intervals (<17 months; 29.2% compared with 11.4%) (69). No difference was reported between the number of children born after long birth intervals who achieved the highest grade in their previous year of schooling compared with those children born after short birth intervals (33.0% compared with 23.4%) (69). Likewise, no difference was reported in the number of children born after long birth intervals which were classified as above average for teacher-assessed school performance compared with those children born after short birth intervals (48% compared with 34%) (69). Furthermore, there was no difference in the number of children born after a long birth interval who scored below average for teacher-assessed school performance compared with children born after a short birth interval (13.6% compared with 14.6%) (69).

### Cognitive ability

Sujan et al. (73) examined the associations between cognitive ability assessed using the Maths, Reading, and Reading Recognition subtests of the PIAT-R and the Digit Span subtest of the WISC-R (73). After adjustment for child's sex and birth order, compared with the reference category (IPIs of >12–36 months), short IPIs (≤12 months) were associated with an increased risk of lower standardized scores on Maths (aOR, 0.78; 95% CI, 0.68–0.90), Reading (aOR, 0.83; 95% CI, 0.73–0.94), and Reading Recognition (aOR, 0.84; 95% CI, 0.74–0.95) subtests (73). After adjustment for child's sex and birth order, compared with the reference category, long IPIs (>36 months) were associated with Maths (aOR, 0.86; 95% CI, 0.77–0.96), only (73). In the fully adjusted models, there was no association between standardized scores on measures of Maths, Digit Span, Reading, and Reading Recognition compared to the reference category (IPIs of >12–36 months) for children born after short or long IPIs (73).

### Cognitive development

Bella et al. (70) reported no difference in the number of children born after short birth intervals (<13 months) who scored average or above average on the Raven's Matrices test compared with children born after long birth intervals of >31 months (86.7% compared with 85.6%) (70). Overall, no difference was reported between the mean Raven's Matrices test scores with respect to birth intervals—however, lower mean Raven's Matrices test scores were reported with increasing birth intervals (70).

### Risk of bias

For observational studies, the risk of bias scores ranged from 3 to 9 on the Newcastle-Ottawa scale, of which four studies (8, 71, 73, 74) were deemed to be at low risk of bias and two studies were deemed to be at moderate (70, 72) risk of bias and one study was deemed to be at high (69) risk of bias (Supplementary Tables S3–S5).

## Discussion

There has been increasing interest in understanding the associations between the time interval between pregnancies and the health and developmental outcomes of children. Several systematic reviews and meta-analyses have reported that sub-optimal IPIs are associated with an increased risk of adverse prenatal and perinatal health outcomes (14, 16, 21, 36, 37); however, these reviews have not examined child development outcomes beyond infancy. Furthermore, we have identified that only a minority of the existing studies have aimed to assess child development outcomes beyond infancy. Consequently, this review highlights the paucity of research examining the effects of birth spacing on child development outcomes into the early childhood period (33). In this systematic review, we summarized the results from seven studies, including information on >65,500 children. A narrative synthesis of the studies included in this review indicates that there is limited evidence evaluating the associations between birth spacing and child development outcomes in the early childhood period. Despite this, this review highlights that the effects of sub-optimal birth spacing are observable beyond infancy.

One of the two cross-sectional studies reported that children born after short birth intervals (<13 months) were more likely to score higher on school performance compared with children born after longer birth intervals (>31 months) (70). The other cross-sectional study also reported no difference in the number of children born after short birth intervals (<17 months) who scored above average in general intelligence on the Raven's Matrices test (70). IPIs of ≤12 months were associated with lower than average scores on measures of Vocabulary, Maths, Reading, and Reading Recognition, after adjustment for child's sex and birth order (73). However, associations between short IPIs and cognitive ability were completely attenuated in the fully adjusted models. Combined, the findings of these studies contribute to the evidence base that the risk of poorer child development outcomes for children born after short IPIs is not equivalent to that of children born after IPIs of >12–36 months (69, 70, 73).

Two additional studies reported that short birth spacing [birth intervals of <24 months (72) and IPIs of <6 and 6–11 months (8)] were associated with poorer child development outcomes. After adjusting for a range of maternal- and child-related factors, birth intervals of <24 months were also associated with poorer school readiness scores in the Cognitive Skills Assessment Battery (72). The same study reported that a 6-month incremental increase in the birth interval was associated with children being less likely to have poorer school readiness, thus suggesting the adverse implications of longer birth intervals are also observable beyond infancy (72). Likewise, short IPIs of <6 months were associated with an increased risk of children being classified as developmentally vulnerable on one or more and two or more AEDC domains (8). Short IPIs of <6 months were also associated with developmental vulnerability in the Physical Health and Wellbeing and Emotional Maturity domains of the AEDC (8).

Findings from studies examining the associations between long birth spacing were mixed. Havron et al. (71) reported that the age gap between siblings was not associated with language skills. IPIs of >36 months were associated with lower than average scores on measures of Maths and Recognition after adjustment for child's sex and birth order (73). However, no association was observed between long IPIs and standardized scores on measures of cognitive ability (73). Dhamrait et al. (8) reported that after adjusting for a range of maternal- and child-related factors, IPIs of ≥24 months were associated with an increased risk of children being classified as developmentally vulnerable on one or more and two or more AEDC domains (8). Furthermore, IPIs of ≥24 months were also associated with an increased risk of developmental vulnerability for the Social Competence and Emotional Maturity domains of the AEDC, whilst IPIs of ≥60 months were associated with an increased risk of developmental vulnerability on all five AEDC domains (8).

Differences in results between the studies may be attributable to differences in sample size and the adjustment variables used in the adjusted models. The studies that reported the greatest effect sizes (8, 72, 74) for the associations between birth spacing and adverse child development outcomes had typically larger sample sizes compared with the studies that reported no association (69, 71) or associations in partially adjusted models (73), only. Whilst the included studies accounted for potential risk factors related to maternal characteristics such as age and child characteristics such as birthweight and gestational age (71, 72), only two studies adjusted for either birth order (73) or parity (8). There is an extensive literature base reporting on the relationship between low birthweight and an increased risk of poorer cognitive and school performance outcomes when compared with normal birthweight peers, and this risk for adverse outcomes increases with decreasing birthweight (75–78). Likewise, studies using US (79) and non-US samples (28–30, 80) have observed a dose-dependent relationship between gestational age and poor child development outcomes at age five. Thus, studies in this review adjusting for birthweight and gestational age provide an underestimate of the total effect of associations between birth spacing and child development.

Nathens et al. (74) compared the effects on risk of injury for children without an older sibling to those children with an older sibling (stratifying by birth intervals between the older sibling and the cohort child). Although this study cannot be compared directly with the studies that investigated the effects of birth spacing between children with older siblings, this study reported that the risk of injury for children decreased with increasing birth interval (74). Overall, given the considerable variation in the definitions of birth spacing across the included studies, further conclusions about the associations between birth intervals and child development outcomes cannot be drawn.

Existing studies have assessed a range of child development outcomes with respect to birth intervals. Compared with IPIs, birth interval measures have an inherent bias (14) as this measure is conflated by the gestational length of the subsequent pregnancy; shorter pregnancy duration is associated with poorer pregnancy outcomes which are further linked to poorer child development outcomes in the early childhood period. Furthermore, more studies with larger sample sizes are required to assess the associations between birth spacing and child development outcomes. In addition, no studies included in this review reported on whether the pregnancies were intended. Likewise, it is evident from the studies included in this review that the existing studies assessing the associations between birth spacing on child development outcomes have assessed a wide range of development outcomes. Thus, differences in study quality, exposure definitions, study outcomes and ascertainment, and definitions of reference categories—which varied from children with no older sibling (74) to birth intervals of ≥24–120 months (72)—made it difficult to compare results across studies and the reliability of current findings.

Our review identified a need for further research investigating the effects of birth spacing, particularly IPIs, on child development outcomes. The categorization of measures of birth spacing was inconsistent, with the definitions of birth spacing between siblings varying significantly across the studies included in this review. Only two studies adjusted for paternal risk factors (8, 71), and only one study used IPIs of 18–23 months as the reference category (8). In summary, we recommend that future studies adopt the following: (1) consistent categorization for measures of pregnancy spacing; (2) use IPIs as a measure for pregnancy spacing; and (3) use of a comprehensive set of pregnancy-, birth-, child- and family-level characteristics. Given the World Health Organization's recommendation of IPIs of approximately 2–3 years to reduce adverse birth outcomes among children (33), and the lowest risk of adverse perinatal outcomes observed for IPIs of 18–23 months (15). Thus, we recommend future studies align with the World Health Organization's recommendation to use IPIs of 18–23 months (equivalent to birth intervals of 27–30 months) as the reference category when investigating the effects of IPI as a categorical variable.

## Conclusion

Evidence from this review, albeit limited, is suggestive of potential adverse effects of both short and long birth spacing between successive pregnancies on childhood development beyond infancy. However, additional studies which adhere to the World Health Organization's recommendations of IPIs of 18–23 months are required to better establish the potential association between IPIs and early childhood development outcomes.

## Data Availability

The original contributions presented in the study are included in the article/Supplementary Material, further inquiries can be directed to the corresponding author/s.
