# Supplementary material for: The effects of birth spacing on early childhood development in high-income nations: A systematic review
Source: Front Pediatr. 2022 Nov 25;10:851700. doi: 10.3389/fped.2022.851700 (PMC9732574; doi:10.3389/fped.2022.851700)
Supplement: Supplementary file 1 [file Datasheet1.docx]

**Appendix One**

**Table A1.** Summary of Search Strategy for Systematic Review of The Effects of Interpregnancy Intervals on Child Development Outcomes: Ovid/Medline Database.

| **No.** | **Searches** | **Results** |
| --- | --- | --- |
| 1. | exp Child Development/ or exp "Early Intervention (Education)"/ or exp Child, Preschool/ or school readiness.mp. | 985511 |
| 2. | exp Child Health/ | 3693 |
| 3. | exp child behavio?r/ | 25186 |
| 4. | Personality Development/ | 12952 |
| 5. | exp language development/ | 16856 |
| 6. | (education* outcome* or education* status).mp. [mp=title, abstract, original title, name of substance word, subject heading word, floating sub-heading word, keyword heading word, organism supplementary concept word, protocol supplementary concept word, rare disease supplementary concept word, unique identifier, synonyms] | 59922 |
| 7. | health outcome*.mp. | 61180 |
| 8. | emotional maturity.mp. | 134 |
| 9. | 1 or 2 or 3 or 4 or 5 or 6 or 7 or | 1120855 |
| 10. | exp birth intervals/ | 1803 |
| 11. | (((Interpregnancy adj2 interval*) or inter-pregnancy or pregnancy) adj2 interval).mp. [mp=title, abstract, original title, name of substance word, subject heading word, floating sub-heading word, keyword heading word, organism supplementary concept word, protocol supplementary concept word, rare disease supplementary concept word, unique identifier, synonyms] | 894 |
| 12. | (((birth spac* or interconception interval or time to birth or birth interval* or interdelivery interval or birth) adj3 interval) or delivery to conception interval).mp. [mp=title, abstract, original title, name of substance word, subject heading word, floating sub-heading word, keyword heading word, organism supplementary concept word, protocol supplementary concept word, rare disease supplementary concept word, unique identifier, synonyms] | 1402 |
| 13. | 10 or 11 or 12 | 3285 |
| 14. | exp siblings/ | 12058 |
| 15. | exp Family Characteristics/ | 67091 |
| 16. | ((sibling number* or sibling count or number of sibling* or sibling* or sibling relation* or sibling*) adj2 number*).mp. [mp=title, abstract, original title, name of substance word, subject heading word, floating sub-heading word, keyword heading word, organism supplementary concept word, protocol supplementary concept word, rare disease supplementary concept word, unique identifier, synonyms] | 1155 |
| 17. | ((family adj2 size) or Size of famil* or family plan*).mp. [mp=title, abstract, original title, name of substance word, subject heading word, floating sub-heading word, keyword heading word, organism supplementary concept word, protocol supplementary concept word, rare disease supplementary concept word, unique identifier, synonyms] | 54729 |
| 18. | (intergenerat* famil* or multigenerat* famil*).mp. [mp=title, abstract, original title, name of substance word, subject heading word, floating sub-heading word, keyword heading word, organism supplementary concept word, protocol supplementary concept word, rare disease supplementary concept word, unique identifier, synonyms] | 716 |
| 19. | 14 or 15 or 16 or 17 or 18 | 128889 |
| 20. | (socioeconomic or sociodemographic or sociocultural or sociological).mp. [mp=title, abstract, original title, name of substance word, subject heading word, floating sub-heading word, keyword heading word, organism supplementary concept word, protocol supplementary concept word, rare disease supplementary concept word, unique identifier, synonyms] | 288806 |
| 21. | family environment.mp. | 3044 |
| 22. | social determinants of health.mp. | 9515 |
| 23. | social class.mp. | 47469 |
| 24. | exp SOCIOECONOMIC FACTORS/ | 472260 |
| 25. | exp Educational Status/ | 54554 |
| 26. | 20 or 21 or 22 or 23 or 24 or 25 | 584214 |
| 27. | 9 and 13 and 19 and 26 | 358 |
| 28. | limit 27 to English language | 335 |
| 29. | limit 28 to yr="1989 -Current" | 262 |

**Table A2.** Summary of Search Strategy for Systematic Review of The Effects of Interpregnancy Intervals on Child Development Outcomes: Global Health Database.

| **No.** | **Searches** | **Results** |
| --- | --- | --- |
| 1. | exp child health/ | 13418 |
| 2. | exp child development/ | 2812 |
| 3. | child behavio$r.mp. | 607 |
| 4. | (education* outcome* or education* status).mp. [mp=abstract, title, original title, broad terms, heading words, identifiers, cabicodes] | 3017 |
| 5. | personality development.mp. | 52 |
| 6. | (((child adj2 education) or child) adj2 wellbeing).mp. [mp=abstract, title, original title, broad terms, heading words, identifiers, cabicodes] | 142 |
| 7. | language development.mp. | 336 |
| 8. | exp preschool children/ | 15230 |
| 9. | 1 or 2 or 3 or 4 or 5 or 6 or 7 or 8 | 33841 |
| 10. | (Interpregnancy interval* or inter-pregnancy or pregnancy interval).mp. [mp=abstract, title, original title, broad terms, heading words, identifiers, cabicodes] | 298 |
| 11. | exp families/ | 17430 |
| 12. | exp family life/ | 720 |
| 13. | exp family size/ | 1408 |
| 14. | exp family structure/ | 772 |
| 15. | (((birth spac* or interconception interval or time to birth or birth interval* or interdelivery interval or birth) adj3 interval) or delivery to conception interval).mp. [mp=abstract, title, original title, broad terms, heading words, identifiers, cabicodes] | 654 |
| 16. | exp siblings/ | 3246 |
| 17. | ((family adj2 size) or family plan*).mp. [mp=abstract, title, original title, broad terms, heading words, identifiers, cabicodes] | 11647 |
| 18. | ((sibling count or sibling* or sibling relation* or sibling*) adj2 number*).mp. [mp=abstract, title, original title, broad terms, heading words, identifiers, cabicodes] | 536 |
| 19. | intergenerat* famil* or multigenerat* famil*).mp. [mp=abstract, title, original title, broad terms, heading words, identifiers, cabicodes] | 54 |
| 20. | (socioeconomic or sociodemographic or sociocultural or sociological).mp. [mp=abstract, title, original title, broad terms, heading words, identifiers, cabicodes] | 89305 |
| 21. | social determinants of health.mp. | 2478 |
| 22. | exp social classes/ | 2327 |
| 23. | exp family environment/ or socioeconomic status.sh. | 37818 |
| 24. | 20 or 21 or 22 or 23 | 93700 |
| 25. | exp infants/ | 150773 |
| 26. | children/ | 392128 |
| 27. | 25 or 26 | 492137 |
| 28. | 10 or 11 or 12 or 13 or 14 or 15 or 16 or 17 or 18 or 19 | 492137 |
| 29. | 9 and 24 and 27 and 28 | 461 |
| 30. | limit 29 to (english language and yr="1989 -Current") | 395 |

**Table A3.** Summary of Search Strategy for Systematic Review of The Effects of Interpregnancy Intervals on Child Development Outcomes: PsycINFO Database.

| **No.** | **Searches** | **Results** |
| --- | --- | --- |
| 1. | exp Childhood Development/ | 111927 |
| 2. | exp Personality Development/ | 22396 |
| 3. | exp Language Development/ | 30542 |
| 4. | (education* outcome* or education* status).mp. [mp=title, abstract, heading word, table of contents, key concepts, original title, tests & measures, mesh] | 20138 |
| 5. | exp School Readiness/ or exp Project Head Start/ or exp Academic Achievement/ or exp Social Skills/ or exp Early Childhood Development/ or exp Kindergarten Students/ | 136155 |
| 6. | exp PRESCHOOL STUDENTS/ or exp PRESCHOOL EDUCATION/ | 16210 |
| 7. | (child health or school readiness).mp. | 10750 |
| 8. | 1 or 2 or 3 or 4 or 5 or 6 or 7 | 283705 |
| 9. | exp Birth Order/ | 2350 |
| 10. | ((interpregnancy adj2 interval*) or inter-pregnancy or pregnancy interval).mp. [mp=title, abstract, heading word, table of contents, key concepts, original title, tests & measures, mesh] | 77 |
| 11. | ((birth spac* or interconception interval or time to birth or birth interval* or interdelivery interval or birth) adj3 interval).mp. [mp=title, abstract, heading word, table of contents, key concepts, original title, tests & measures, mesh] | 159 |
| 12. | exp Family Planning/ | 13861 |
| 13. | 9 or 10 or 11 or 12 | 16376 |
| 14. | exp SIBLINGS/ | 15261 |
| 15. | exp Family Size/ | 1174 |
| 16. | exp Family Structure/ | 16135 |
| 17. | (intergenerat* famil* or multigenerat* famil*).mp. [mp=title, abstract, heading word, table of contents, key concepts, original title, tests & measures, mesh] | 910 |
| 18. | 14 or 15 or 16 or 17 | 31494 |
| 19. | exp Family Background/ | 9466 |
| 20. | exp Socioeconomic Status/ | 60517 |
| 21. | exp Sociocultural Factors/ | 123897 |
| 22. | exp "Quality of Life"/ | 45975 |
| 23. | exp Parent Child Relations/ or exp Parent Educational Background/ | 71930 |
| 24. | exp Demographic Characteristics/ | 184047 |
| 25. | exp "Income (Economic)"/ or exp Income Level/ | 22531 |
| 26. | exp "Racial and Ethnic Differences"/ or exp Demographic Characteristics/ | 218774 |
| 27. | 19 or 20 or 21 or 22 or 23 or 24 or 25 or 26 | 445177 |
| 28. | 8 and 13 and 18 and 27 | 157 |
| 29. | limit 59 to (english language and yr=""1989 -Current") | 64 |

**Table A4.** Summary of Search Strategy for Systematic Review of The Effects of Interpregnancy Intervals on Child Development Outcomes: EMBASE.

| **No.** | **Searches** | **Results** |
| --- | --- | --- |
| 1. | exp child health/ | 29924 |
| 2. | exp child development/ | 46634 |
| 3. | exp child behavio?r/ | 51304 |
| 4. | school readiness.mp. | 733 |
| 5. | exp language development/ | 16248 |
| 6. | (education* outcome* or education* status or health outcome*).mp. [mp=title, abstract, heading word, drug trade name, original title, device manufacturer, drug manufacturer, device trade name, keyword, floating subheading word, candidate term word] | 165554 |
| 7. | exp early childhood intervention/ | 2998 |
| 8. | 1 or 2 or 3 or 4 or 5 or 6 or 7 | 297247 |
| 9. | exp family planning/ | 36011 |
| 10. | (((Interpregnancy adj2 interval*) or inter-pregnancy or pregnancy) adj2 interval).mp. [mp=title, abstract, heading word, drug trade name, original title, device manufacturer, drug manufacturer, device trade name, keyword, floating subheading word, candidate term word] | 1281 |
| 11. | (((birth spac* or interconception interval or time to birth or interdelivery interval or birth*) adj3 interval*) or delivery to conception interval).mp. [mp=title, abstract, heading word, drug trade name, original title, device manufacturer, drug manufacturer, device trade name, keyword, floating subheading word, candidate term word] | 2586 |
| 12. | 9 or 10 or 11 | 38660 |
| 13. | exp siblings/ | 48262 |
| 14. | exp Family Characteristics/ | 18880 |
| 15. | exp family relations/ | 107060 |
| 16. | (family adj2 size).mp. [mp=title, abstract, heading word, drug trade name, original title, device manufacturer, drug manufacturer, device trade name, keyword, floating subheading word, candidate term word] | 20899 |
| 17. | ((sibling count or sibling* or sibling relation* or sibling*) adj2 number*).mp. [mp=title, abstract, heading word, drug trade name, original title, device manufacturer, drug manufacturer, device trade name, keyword, floating subheading word, candidate term word] | 1446 |
| 18. | (intergenerat* famil* or multigenerat* famil*).mp. [mp=title, abstract, heading word, drug trade name, original title, device manufacturer, drug manufacturer, device trade name, keyword, floating subheading word, candidate term word] | 878 |
| 19. | 13 or 14 or 15 or 16 or 17 or 18 | 172694 |
| 20. | (socioeconomic or sociodemographic or sociocultural or sociological).mp. [mp=title, abstract, heading word, drug trade name, original title, device manufacturer, drug manufacturer, device trade name, keyword, floating subheading word, candidate term word] | 206216 |
| 21. | family environment.mp. | 3989 |
| 22. | social determinants of health.mp. | 14202 |
| 23. | social class.mp. | 36403 |
| 24. | exp socioeconomics/ | 412710 |
| 25. | 20 or 21 or 22 or 23 or 24 | 583713 |
| 26. | 8 and 12 and 19 and 25 | 369 |
| 27. | limit 26 to english language | 329 |
| 28. | limit 27 to yr="1989 -Current" | 213 |

**Table A5.** Summary of Search Strategy for Systematic Review of The Effects of Interpregnancy Intervals on Child Development Outcomes: CINAHL Plus.

| **No.** | **Searches** | **Results** |
| --- | --- | --- |
| S25 | S7 AND S11 AND S17 AND S24  Limiters - Published Date: 19890101-20210631; Language: English | 248 |
| S24 | S18 OR S19 OR S20 OR S21 OR S22 OR S23 | 429,737 |
| S23 | parent* N2 education | 13,752 |
| S22 | (MH "Income+") | 57,558 |
| S21 | socioeconomic OR sociodemographic OR sociocultural OR sociological OR socio-economic status | 148,773 |
| S20 | (MH "Social Class") | 12,322 |
| S19 | (MH "Social Determinants of Health") | 7,356 |
| S18 | (MH "Socioeconomic Factors+") | 371,086 |
| S17 | S12 OR S13 OR S14 OR S15 OR S16 | 48,444 |
| S16 | (MH "Siblings") | 3,725 |
| S15 | sibling count or sibling relation* or sibling* N2 number* | 1,433 |
| S14 | intergenerat* famil* or multigenerat* famil* | 6,207 |
| S13 | family N2 size | 2,259 |
| S12 | (MH "Family Characteristics+") | 728 |
| S11 | S8 OR S9 OR S10 | 1,114 |
| S10 | birth spac* or interconception interval or time to birth or interdelivery interval or birth N3 interval* | 39,841 |
| S9 | Interpregnancy N2 interval* or inter-pregnancy or pregnancy N2 interval | 2,451 |
| S8 | (MH "Birth Order") OR (MH "Birth Intervals") | 753 |
| S7 | S1 OR S2 OR S3 OR S4 OR S5 OR S6 | 176,595 |
| S6 | (MH "Language Development") OR MH "Early Childhood Intervention") OR (MH "Learning Environment+") | 28,383 |
| S5 | education* N2 status or education* N2 outcome* OR educational measurement OR education* N2 measure* | 77,501 |
| S4 | school N3 read* OR academic readiness OR academic N3 read* | 1,489 |
| S3 | Child Behavior | 36,507 |
| S2 | (MH "Child Health") | 16,601 |
| S1 | (MH "Child Development") OR "child development" | 30,609 |

**Table A6.** Summary of Search Strategy for Systematic Review of The Effects of Interpregnancy Intervals on Child Development Outcomes: Educational Source.

| **No.** | **Searches** | **Results** |
| --- | --- | --- |
| S24 | S7 AND S12 AND S18 AND S23 | 3 |
| S23 | (S19 OR S20 OR S21 OR S22) | 104377 |
| S22 | household income | 786 |
| S21 | socioeconomic OR sociodemographic OR sociocultural OR sociological OR socioeconomic status | 8640 |
| S20 | DE "Education of parents" | 96409 |
| S19 | DE "Socioeconomically disadvantaged students" | 246 |
| S18 | (S13 OR S14 OR S15 OR S16 OR S17) | 6253 |
| S17 | sibling count or sibling relation* or sibling* N2 number* | 3477 |
| S16 | intergenerat* famil* or multigenerat* famil* | 828 |
| S15 | (DE "Family demography") | 124 |
| S14 | DE "Siblings" OR DE "Brothers" | 4274 |
| S13 | DE "Family size" | 575 |
| S12 | (S8 OR S9 OR S10 OR S11) | 1815 |
| S11 | DE "Birth date effect (Academic achievement)" | 11 |
| S10 | birth N2 spac* or time to birth or interdelivery interval or birth N3 interval* | 1239 |
| S9 | Interpregnancy N2 interval* or inter-pregnancy or pregnancy N2 interval* | 29 |
| S8 | DE "Birth order" | 612 |
| S7 | (S1 OR S2 OR S3 OR S4 OR S5 OR S6) | 82183 |
| S6 | DE "Cognitive development" OR DE "Class inclusion (Child psychology)" | 7853 |
| S5 | DE "Emotional intelligence" OR DE "Emotional maturity" | 3687 |
| S4 | DE "Children's language" OR DE "Baby signing (Sign language)" OR DE "Language Environment Analysis System" | 8897 |
| S3 | DE "Children's health" OR DE "Child nutrition" OR DE "Physical education for children" | 13622 |
| S2 | DE "Child development" OR DE "Asynchronous child development" OR DE "Child psychology" OR DE "Developmental delay" OR DE "Motor ability in children" OR DE "Play groups" OR DE "Child development testing" | 50728 |
| S1 | DE "Readiness for school" OR DE "Readiness for school research" OR DE "Readiness for school testing" OR DE "Gesell School Readiness Test" OR DE "Lollipop Test (Readiness for school)" OR DE "Preschool tests" | 2139 |

**Table A7.** Summary of Search Strategy for Systematic Review of The Effects of Interpregnancy Intervals on Child Development Outcomes: Research Starters.

| S12 | S4 AND S5 AND S8 AND S11 | 2 |
| --- | --- | --- |
| S11 | S9 OR S10 | 522 |
| S10 | (Socioeconomic status or poverty or low income) OR socioeconomic factors | 299 |
| S9 | (Socioeconomic OR sociodemographic OR sociocultural OR sociological OR socio-economic status) OR (household income OR parent* N2 income) OR parent* N2 education | 330 |
| S8 | S6 OR S7 | 66 |
| S7 | (Family characteristic* OR family structure OR (sibling* OR brother* OR sister*) | 63 |
| S6 | family N2 size OR (intergenerat* famil* OR multigenerat* famil*) OR (sibling count OR sibling relation* OR sibling* N2 number* ) | 21 |
| S5 | (Birth N2 order OR Birth N2 intervals) OR pregnancy interval OR (birth spac* OR interconception interval OR time to birth OR interdelivery interval OR birth N3 interval*) | 28 |
| S4 | S1 OR S2 OR S3 | 1,072 |
| S3 | educational measurement | 189 |
| S2 | (Education* N2 status or education* N2 outcome*) OR (language Development OR Early Childhood Intervention OR Learning Environment) OR (child N2 behavior OR child N2 behaviour) | 568 |
| S1 | (Child* N3 Development OR Child Health OR (school N2 read* OR academic* N2 read* OR school readiness) | 438 |

**Table A8.** Summary of Search Strategy for Systematic Review of The Effects of Interpregnancy Intervals on Child Development Outcomes: ERIC.

| **Searches** | **Results** |
| --- | --- |
| ((MAINSUBJECT.EXACT.EXPLODE("School Readiness") OR MAINSUBJECT.EXACT.EXPLODE("Preschool Children") OR MAINSUBJECT.EXACT("Preschool Learning") OR MAINSUBJECT.EXACT.EXPLODE("Preschool Education") OR MAINSUBJECT.EXACT.EXPLODE("Child Language") OR MAINSUBJECT.EXACT.EXPLODE("Child Welfare") OR MAINSUBJECT.EXACT.EXPLODE("Child Health") OR MAINSUBJECT.EXACT.EXPLODE("Child Development") OR MAINSUBJECT.EXACT.EXPLODE("Child Behavior") OR MAINSUBJECT.EXACT.EXPLODE("Language Acquisition")) AND (MAINSUBJECT.EXACT.EXPLODE("Siblings") OR MAINSUBJECT.EXACT.EXPLODE("Parent Child Relationship") OR MAINSUBJECT.EXACT.EXPLODE("Family Characteristics") OR MAINSUBJECT.EXACT.EXPLODE("Sibling Relationship") OR MAINSUBJECT.EXACT.EXPLODE("Birth Order") OR MAINSUBJECT.EXACT("Economic Factors") OR MAINSUBJECT.EXACT.EXPLODE("Socioeconomic Background") OR MAINSUBJECT.EXACT.EXPLODE("Parent Education") OR MAINSUBJECT.EXACT.EXPLODE("Educationally Disadvantaged") OR MAINSUBJECT.EXACT.EXPLODE("Socioeconomic Status") OR MAINSUBJECT.EXACT("Social Influences") OR MAINSUBJECT.EXACT.EXPLODE("Parent Background") OR MAINSUBJECT.EXACT.EXPLODE("Social Class") OR MAINSUBJECT.EXACT.EXPLODE("Gifted Disadvantaged") OR MAINSUBJECT.EXACT.EXPLODE("Economically Disadvantaged") OR MAINSUBJECT.EXACT.EXPLODE("Disadvantaged Youth")) AND (MJMESH.EXACT("Birth Intervals") OR MAINSUBJECT.EXACT.EXPLODE("Family Planning") OR MAINSUBJECT.EXACT.EXPLODE("Family Size"))) AND rtype.exact("080: Journal Articles" OR "080 Journal Articles" OR "Article") AND la.exact("English") AND pd(>19890625) | 46 |

**Table A9.** Summary of Search Strategy for Systematic Review of The Effects of Interpregnancy Intervals on Child Development Outcomes: ProQuest’s Social Sciences Database.

| **Searches** | **Results** |
| --- | --- |
| (((MAINSUBJECT.EXACT("School Readiness") OR MAINSUBJECT.EXACT("Preschool Children") OR MAINSUBJECT.EXACT("Preschool Learning") OR MAINSUBJECT.EXACT("Preschool Education") OR MAINSUBJECT.EXACT("Child Language") OR MAINSUBJECT.EXACT("Child Welfare") OR MAINSUBJECT.EXACT("Child Health") OR MAINSUBJECT.EXACT("Child Development") OR MAINSUBJECT.EXACT("Child Behavior") OR MAINSUBJECT.EXACT("Language Acquisition")) AND (MAINSUBJECT.EXACT("Siblings") OR MAINSUBJECT.EXACT("Parent Child Relationship") OR MAINSUBJECT.EXACT("Family Size") OR MAINSUBJECT.EXACT("Family Characteristics") OR MAINSUBJECT.EXACT("Sibling Relationship") OR MAINSUBJECT.EXACT("Birth Order") OR MAINSUBJECT.EXACT("Economic Factors") OR MAINSUBJECT.EXACT("Socioeconomic Background") OR MAINSUBJECT.EXACT("Parent Education") OR MAINSUBJECT.EXACT("Educationally Disadvantaged") OR MAINSUBJECT.EXACT("Socio economic Status") OR MAINSUBJECT.EXACT("Social Influences") OR MAINSUBJECT.EXACT("Parent Background") OR MAINSUBJECT.EXACT("Social Class") OR MAINSUBJECT.EXACT("Gifted Disadvantaged") OR MAINSUBJECT.EXACT("Family Planning") OR MAINSUBJECT.EXACT("Economically Disadvantaged") OR MAINSUBJECT.EXACT("Disadvantaged Youth")) AND Quantitative OR quantitative study OR randomized controlled trial OR non- randomized controlled trial OR experimental OR quasi- experimental OR observational study OR case-control OR cohort OR descriptive OR case series OR cross sectional OR clinical trial)) AND MAINSUBJECT.EXACT("Preschool Children")) AND (at.exact("Article") AND stype.exact("Scholarly Journals") AND la.exact("ENG") AND pd(>19891231) | 152 |
