# Supplementary material for: The effects of birth spacing on early childhood development in high-income nations: A systematic review
Source: Front Pediatr. 2022 Nov 25;10:851700. doi: 10.3389/fped.2022.851700 (PMC9732574; doi:10.3389/fped.2022.851700)
Supplement: Supplementary file 2 [file Datasheet2.docx]

**Supplemental Table 1.** Names of databases included in ProQuest’s Social Sciences Database.

| 1. Australian & New Zealand Database |
| --- |
| 1. Biology Database |
| 1. East Europe, Central Europe Database |
| 1. Education Database |
| 1. Continental Europe Database |
| 1. Family Health Database |
| 1. Health & Medical Collection |
| 1. Health Management Database |
| 1. India Database |
| 1. Latin America & Iberia Database |
| 1. Library Science Database |
| 1. Linguistic Database |
| 1. Middle East & Africa Database |
| 1. Nursing & Allied Health Database |
| 1. Political Science Database |
| 1. Psychology Database |
| 1. Public Health Database |
| 1. Research Library |
| 1. Science Database |
| 1. Social Sciences Database |
| 1. SciTech Premium Collection – Science Database |
| 1. Sociology Database |
| 1. Turkey Database |
| 1. UK & Ireland Database |

**Supplemental Table 2.** Summary of the Master Search Strategy for Systematic Review of The Effects of Birth Spacing on Early Childhood Development in High-Resource Settings.

| **No.** | **Searches** |
| --- | --- |
| 1. | exp Child Development/ or exp "Early Intervention (Education)"/ or exp Child, Preschool/ or school readiness.mp. |
| 2. | exp Child Health/ |
| 3. | exp child behavio?r/ |
| 4. | Personality Development/ |
| 5. | exp language development/ |
| 6. | (education* outcome* or education* status).mp. [mp=title, abstract, original title, name of substance word, subject heading word, floating sub-heading word, keyword heading word, organism supplementary concept word, protocol supplementary concept word, rare disease supplementary concept word, unique identifier, synonyms] |
| 7. | health outcome*.mp. |
| 8. | emotional maturity.mp. |
| 9. | 1 or 2 or 3 or 4 or 5 or 6 or 7 or |
| 10. | exp birth intervals/ |
| 11. | (((Interpregnancy adj2 interval*) or inter-pregnancy or pregnancy) adj2 interval).mp. [mp=title, abstract, original title, name of substance word, subject heading word, floating sub-heading word, keyword heading word, organism supplementary concept word, protocol supplementary concept word, rare disease supplementary concept word, unique identifier, synonyms] |
| 12. | (((birth spac* or interconception interval or time to birth or birth interval* or interdelivery interval or birth) adj3 interval) or delivery to conception interval).mp. [mp=title, abstract, original title, name of substance word, subject heading word, floating sub-heading word, keyword heading word, organism supplementary concept word, protocol supplementary concept word, rare disease supplementary concept word, unique identifier, synonyms] |
| 13. | 10 or 11 or 12 |
| 14. | exp siblings/ |
| 15. | exp Family Characteristics/ |
| 16. | ((sibling number* or sibling count or number of sibling* or sibling* or sibling relation* or sibling*) adj2 number*).mp. [mp=title, abstract, original title, name of substance word, subject heading word, floating sub-heading word, keyword heading word, organism supplementary concept word, protocol supplementary concept word, rare disease supplementary concept word, unique identifier, synonyms] |
| 17. | ((family adj2 size) or Size of famil* or family plan*).mp. [mp=title, abstract, original title, name of substance word, subject heading word, floating sub-heading word, keyword heading word, organism supplementary concept word, protocol supplementary concept word, rare disease supplementary concept word, unique identifier, synonyms] |
| 18. | (intergenerat* famil* or multigenerat* famil*).mp. [mp=title, abstract, original title, name of substance word, subject heading word, floating sub-heading word, keyword heading word, organism supplementary concept word, protocol supplementary concept word, rare disease supplementary concept word, unique identifier, synonyms] |
| 19. | 14 or 15 or 16 or 17 or 18 |
| 20. | (socioeconomic or sociodemographic or sociocultural or sociological).mp. [mp=title, abstract, original title, name of substance word, subject heading word, floating sub-heading word, keyword heading word, organism supplementary concept word, protocol supplementary concept word, rare disease supplementary concept word, unique identifier, synonyms] |
| 21. | family environment.mp. |
| 22. | social determinants of health.mp. |
| 23. | social class.mp. |
| 24. | exp SOCIOECONOMIC FACTORS/ |
| 25. | exp Educational Status/ |
| 26. | 20 or 21 or 22 or 23 or 24 or 25 |
| 27. | 9 and 13 and 19 and 26 |
| 28. | limit 27 to English language |
| 29. | limit 28 to yr="1989 -Current" |

**Supplemental Table 3.** Risk of Bias Assessment Results for Cohort Studies^1^

| **Cohort Studies** | **Overall Risk of Bias Score^2^**  (score out of 9) | **Risk of Bias** | **Criteria** | | | | | | | |
| --- | --- | --- | --- | --- | --- | --- | --- | --- | --- | --- |
|  |  |  | **Selection** | | | | **Comparability** | **Outcome** | | |
|  |  |  | Representativeness of the exposed Cohort | Selection of the non-exposed cohort | Ascertainment of cases | Outcome of interest was not present at the start of study | Comparability of the design and analysis for cohorts | Assessment of outcome | Sufficient follow-up duration | Adequacy of follow-up of cohorts |
| Dhamrait et al. 2021 | 9 | Low | X | X | X | X | XX | X | X | X |
| Havron et al. 2019 | 7 | Low | — | X | X | X | XX | X | X | — |
| Hayes et al. 2006 | 6 | Moderate | — | X | X | X | X | X | X | — |
| Sujan et al. 2019 | 8 | Low | X | X | X | X | X | X | X | X |

^1^Cohort studies were assessed using the Newcastle-Ottawa scale.

^2^Low risk of bias: overall risk of bias score ≥7; moderate risk of bias: overall risk of bias score 4-6; high risk of bias: overall risk of bias score ≤3.

Each X represents whether an individual criterion is satisfied.

Each — represents whether an individual criterion is not satisfied.

**Supplemental Table 4.** Risk of Bias Assessment Results for Case-Control Studies.^1^

| **Case-control Studies** | **Overall Risk of Bias Score^2^**  (score out of 9) | **Risk of Bias** | **Criteria** | | | | | | | |
| --- | --- | --- | --- | --- | --- | --- | --- | --- | --- | --- |
|  |  |  | **Selection** | | | | **Comparability** | **Outcome** | | |
|  |  |  | Definition of cases | Representativeness of the exposed cases | Adequacy of case definitions | Definition of controls | Comparability of the design and analysis for cohorts | Ascertainment of exposure | Comparability of ascertainment of exposure for cohorts | Comparability of non-response rate of cohorts |
| Nathens et al. 2000 | 9 | Low | X | X | X | X | XX | X | X | X |

^1^Case-control studies were assessed using the Newcastle-Ottawa scale.

^2^Low risk of bias: overall risk of bias score ≥7; moderate risk of bias: overall risk of bias score 4-6; high risk of bias: overall risk of bias score ≤3.

Each X represents whether an individual criterion is satisfied.

Each — represents whether an individual criterion is not satisfied.

**Supplemental Table 5.** Risk of Bias Assessment Results for Cross-Sectional Studies^1^.

| **Cross-Sectional Studies** | **Overall Risk of Bias Score^2^**  (score out of 9) | **Risk of Bias** | **Criteria** | | | | | | |
| --- | --- | --- | --- | --- | --- | --- | --- | --- | --- |
|  |  |  | **Selection** | | | | **Comparability** | **Outcome** | |
|  |  |  | Representativeness of the sample | Sample size | Non-respondents | Ascertainment of exposure | Comparability of cohorts on the basis of the design or analysis | Assessment of outcome | Statistical test |
| Bella & Al-Almaie 2005 | 5 | Moderate | — | X | X | XX | — | X | — |
| Bella et al. 2005 | 3 | High | — | — | — | XX | — | X | — |

^1^Case-Sectional studies were assessed using the Newcastle-Ottawa scale.

^2^Low risk of bias: overall risk of bias score ≥7; moderate risk of bias: overall risk of bias score 4-6; high risk of bias: overall risk of bias score ≤3.

Each X represents whether an individual criterion is satisfied.

Each — represents whether an individual criterion is not satisfied.

**Supplemental Table 6.** Exclusion Criteria listed in original studies.

| **Exclusion criteria** | **Author (Year)** | | | | | | |
| --- | --- | --- | --- | --- | --- | --- | --- |
|  | Bella & Al-Almaie (2005) | Bella et al. (2005) | Dhamrait et al. (2021) | Havron et al. (2019) | Hayes et al. (2006) | Nathens et al. (2000) | Sujan et al. (2019) |
| First-born children and/or children with missing or undeterminable birth intervals | X | X | X | X | X | ⎯ | X |
| Birth order | X^1^ | X^1^ | ⎯ | ⎯ | ⎯ | ⎯ | X^2^ |
| Multiple births | X | X | X | X | X | X | X |
| Children enrolled in special needs schools | X | X | ⎯ | ⎯ | ⎯ | ⎯ | ⎯ |
| Stepsiblings/half-siblings (father’s side) | X | X | ⎯ | ⎯ | ⎯ | ⎯ | ⎯ |
| Children enrolled in private schools | X | X | ⎯ | ⎯ | ⎯ | ⎯ | ⎯ |
| Children identified as being ‘special needs’ based on a diagnosed physical and/or intellectual disability | ⎯ | ⎯ | X | ⎯ | ⎯ | ⎯ | ⎯ |
| Had missing or invalid Australian Early Development (AEDC) scores | ⎯ | ⎯ | X | ⎯ | ⎯ | ⎯ | ⎯ |
| History of diabetes | ⎯ | ⎯ | ⎯ | X | ⎯ | ⎯ | ⎯ |
| Congenital abnormalities/ abnormal newborn conditions | ⎯ | ⎯ | X | ⎯ | X | ⎯ | ⎯ |
| Children with negative or missing interpregnancy intervals | ⎯ | ⎯ | X | ⎯ | ⎯ | ⎯ | X |
| Intention to deliver children outside a specific hospital | ⎯ | ⎯ | ⎯ | X | ⎯ | ⎯ | ⎯ |
| Intention to move out of the study area during the follow-up period | ⎯ | ⎯ | ⎯ | X | ⎯ | ⎯ | ⎯ |
| Inability to speak French | ⎯ | ⎯ | ⎯ | X | ⎯ | ⎯ | ⎯ |
| Maternal ethnicity (mother was not White, non-Hispanic or African American) | ⎯ | ⎯ | ⎯ | ⎯ | X | ⎯ | ⎯ |
| Maternal age (mother was not aged between 20-44 years at time of index pregnancy) | ⎯ | ⎯ | ⎯ | ⎯ | X | ⎯ | ⎯ |
| Birth interval >120 months | ⎯ | ⎯ | ⎯ | ⎯ | X | ⎯ | ⎯ |
| Injuries due to certain ICD-9 codes^3^ | ⎯ | ⎯ | ⎯ | ⎯ | ⎯ | X | ⎯ |
| Children with missing gestational age at birth, small for gestational age at birth or implausible gestational age at birth <23 weeks | ⎯ | ⎯ | ⎯ | ⎯ | ⎯ | ⎯ | X |
| Children with a birthweight <300 grams. | ⎯ | ⎯ | ⎯ | ⎯ | ⎯ | ⎯ | X |

^1^Excluded children if birth order was ≥5.

^2^Excluded children if birth order was ≥6.

^3^ICD-9 codes: E870-E879, E930-E949, E970-E979, and E990-E999.

Each X represents whether an individual criterion is satisfied.

Each — represents whether an individual criterion is not satisfied.
